# *TERT* Promoter Mutations Increase Sense and Antisense Transcription from the *TERT* Promoter

**DOI:** 10.3390/biomedicines9121773

**Published:** 2021-11-26

**Authors:** François Hafezi, Lisa Jaxel, Morgane Lemaire, Jonathan D. Turner, Danielle Perez-Bercoff

**Affiliations:** 1 Department of Infection and Immunity, Luxembourg Institute of Health, 29, Rue Henri Koch, L-4354 Esch-sur-Alzette, Luxembourg; francois.hafezi@lih.lu (F.H.); lisa.jaxel@hotmail.fr (L.J.); morgane.lemaire@lih.lu (M.L.); 2 Faculty of Science, Technology and Medicine, University of Luxembourg, L-4365 Esch-sur-Alzette, Luxembourg; 3 Immune Endocrine and Epigenetics Research Group, Department of Infection and Immunity, Luxembourg Institute of Health, 29, Rue Henri Koch, L-4354 Esch-sur-Alzette, Luxembourg; jonathan.turner@lih.lu; 4 HIV Clinical and Translational Research Group, Department of Infection and Immunity, Luxembourg Institute of Health, 29, Rue Henri Koch, L-4354 Esch-sur-Alzette, Luxembourg

**Keywords:** *TERT* promoter mutations, Telomerase, TERT, *TERT* transcription, bidirectional promoter, lncRNA *TAPAS*

## Abstract

**Background:** Chief among mechanisms of telomerase reverse transcriptase (TERT) reactivation is the appearance of mutations in the *TERT* promoter. The two main *TERT* promoter mutations are C>T transitions located −146C>T and −124C>T upstream from the translational start site. They generate a novel Ets/TCF binding site. Both mutations are mutually exclusive and −124C>T is strikingly overrepresented in most cancers. We investigated whether this mutational bias and mutual exclusion could be due to transcriptional constraints. **Methods:** We compared sense and antisense transcription of a panel of *TERT* promoter-*luciferase* vectors harboring the −124C>T and -146C>T mutations alone or together. lncRNA *TAPAS* levels were measured by RT-PCR. **Results:** Both mutations generally increased *TERT* transcription by 2–4-fold regardless of upstream and downstream regulatory elements. The double mutant increased transcription in an additive fashion, arguing against a direct transcriptional constraint. The −146C>T mutation, alone or in combination with −124C>T, also unleashed antisense transcription. In line with this finding, lncRNA *TAPAS* was higher in cells with mutated *TERT* promoter (T98G and U87) than in cells with wild-type promoter, suggesting that lncRNA *TAPAS* may balance the effect of *TERT* promoter mutations. **Conclusions**: −146C>T and −124C>T *TERT* promoter mutations increase *TERT* sense and antisense transcription, and the double mutant features higher transcription levels. Increased antisense transcription may contain *TERT* expression within sustainable levels.

## 1. Introduction

Telomeres are DNA–protein complexes which act as protective caps for chromosomal ends. They consist of tandem repeats of the sequence TTAGGG bound by Shelterin enzymes. They shield chromosomal ends from inappropriate DNA damage responses, end-to-end fusion events and erosion, maintaining genomic integrity [1,2,3,4]. Telomeres progressively shorten with each cell division. Critically short telomeres which are no longer bound by Shelterins are recognized as double stranded DNA breaks (DSBs), leading to the activation of DNA damage responses, permanent growth arrest, and apoptosis. Therefore, telomeric erosion ensures a finite number of cell divisions [5]. Cancer cells override senescence and apoptosis by restoring telomeres [6,7]. The main mechanism of telomere maintenance (85–90% of cancers) is reactivation of Telomerase, the enzyme that extends telomeres [8,9,10,11] and particularly of its reverse transcriptase subunit (TERT) [8,10,11,12]. 

*TERT* transcription is tightly regulated. Promoter characterization studies have localized the core promoter within 300 base pairs (bp) uptream from the translational start site (TSS). The *TERT* core promoter is essential for transcriptional activation as it harbors binding sites for numerous activating transcription factors including c-Myc (E-box), Sp1, HIF-1α, STAT, Ets/CTF, and HSP90 [10,13,14]. Binding of CTCF downstream from the core promoter to the region spanning exon 1, intron 1 and the beginning of exon 2 impairs transcription [15,16]. The proximal upstream region (positions −600 to −300 from the TSS) plays a role in regulation and through methylation [15,17]. Hypomethylation of the *TERT* hypermethylated oncological region (THOR, position −649 to −217 from the TSS) allows binding of transcription factors and is associated with *TERT* reactivation in cancer cell lines [17,18]. Others in contrast report that unmethylated THOR represses *TERT* promoter activity [19]. Finally, the distal upstream promoter region (nucleotides −1800 to −800 from the TSS) contains elements involved in the regulation of alternative splicing of *TERT* mRNA [15]. Like many promoters lacking a TATA- or a CAAT-box and initiator elements, the *TERT* promoter is bidirectional and antisense activity is silenced by the upstream proximal promoter region [15]. Antisense transcription leads to a long non-coding RNA (lncRNA) named *TAPAS*, which has been suggested to negatively regulate *TERT* expression in a negative feedback loop [20]. 

In 31% of cancers, *TERT* reactivation is due to mutations in the *TERT* promoter [21]. The most common *TERT* promoter mutations are located at positions 1295228 (C228T) and 1295250 (C250T) on chromosome 5 and generate C>T transitions −124 and −146 base pairs upstream from the *TERT* TSS. They are highly prevalent in primary gliomas and glioblastoma multiforme (GBM), oligodendrogliomas and astrocytomas [11,22,23,24,25,26,27,28,29,30,31,32,33,34,35,36,37,38,39,40], melanomas, cutaneous basal cell carcinoma and squamous cell carcinoma (SCC) [23,41,42,43,44,45,46,47,48,49], myxoid liposarcomas [22], urothelial bladder carcinoma [24,32,41,50,51,52], hepatocellular carcinoma (HCC) [32,41,53,54,55,56], thyroid cancers [57,58,59,60,61,62,63,64,65], as well as oral and cervical SCC [32,66,67]. Other less frequent tandem mutations at positions −125/−124 CC>TT and −139/−138 CC>TT or germline mutations at position −57C>T are frequently found in cutaneous tumors [44,49]. For unknown reasons, the −124C>T mutation is strongly overrepresented in all cancers, with the exception of skin cancers, where both hotspots are mutated with comparable frequencies [21].

Both mutations generate identical sequences and increase *TERT* transcription by ~2–6 fold by qRT-PCR and reporter assays in vitro [41,68] and in patient tumors [23,25,26,27,28,29,31,41,67,68,69]. They generate novel binding sites (GGAA, or TTCC on the opposite strand) for the Ets/TCF transcription factor family [70,71,72]. Among the 30 members of this transcription factor family, GABP and Ets-1/2 bind *TERT* promoter mutations. GABP selectively binds the −124C>T and −146C>T mutations in GBM, melanoma, and urothelial bladder cancer cell lines [70,71,72]. Unlike the other Ets/TCF family members, GABP is a tetramer composed of GABPA and GABPB dimers. To bind, it needs two nearby in-phase GGAA sites [73,74,75,76] positioned 1, 2, or n helical turns away from each other [70], or brought close together by DNA looping [71]. Ets-1/2 bind the −146C>T mutation and activate transcription in conjunction with the non-canonical NF-κB p52 [69]. *TERT* promoter mutations have been associated with epigenetically active chromatin [18,27,70,77,78]. 

*TERT* promoter mutations and *TERT* reactivation are generally monoallelic [18,27], suggesting that *TERT* reactivation on one allele is sufficient to counteract telomere attrition in cancer cells [27]. Furthermore, *TERT* promoter mutations are mutually exclusive [21,41]. They are also generally absent from cancers where *TERT* transcription is reactivated by other mechanisms such as copy number duplications [79,80] or viral oncogenes [53,54,55,66,67,79,81,82,83,84] reviewed in [21,80]. The reasons why *TERT* reactivation tends to rely on one single mechanism remain elusive. In this work, we asked whether a transcriptional constraint could restrict the appearance of both mutations concomitantly. To address this question, we engineered a series of *TERT* promoter-*luciferase* reporter constructs spanning different upstream and downstream regulatory regions of the *TERT* promoter and carrying the −124C>T and −146C>T mutations alone or in combination (−124C>T + −146C>T). We found that the −124C>T and −146C>T mutations lead to similar increases in transcription, in line with previous reports [41,68] and that both mutations together increase transcription in an additive fashion. Moreover, antisense transcription was increased by the −146C>T mutation alone or in combination with −124C>T. As antisense transcription leads to overexpression of the inhibitory lncRNA *TAPAS* [20], the −146C>T mutation, alone or in combination with −124C>T, may lead to an overall lower level of TERT, explaining its lower prevalence and the absence of the double mutant in vivo.

## 2. Materials and Methods

### 2.1. Cell Lines and Cell Culture

All cell lines (HEK293T, HeLa, T98G and U87) were maintained in DMEM supplemented with 10% fetal calf serum (FCS) (Lonza, Verviers, Belgium), 2 mM L-Glutamine, 50 µg/mL Penicillin and 50 µg/mL Streptomycin (all from Invitrogen, Merelbeke, Belgium).

### 2.2. Construction of TERT Promoter-Luciferase Reporter Expression Vectors

**Figure 1:**
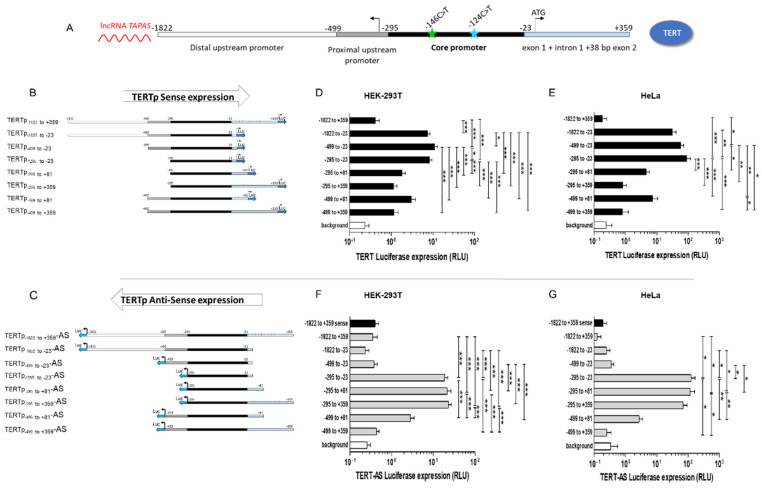
***TERT* transcription regulation.** (**A**). **Schematic representation of the *TERT* promoter.** The *TERT* promoter comprises the core promoter (−295 to −23 from the TSS (black) and upstream and downstream regulatory regions. The TSS is represented with an arrow. Mutations at position −146 and −124 from the TSS are represented by a green and blue star, respectively. (**B**). **Schematic representation of the TERTp-luciferase reporter plasmids in sense orientation.** Different portions of the *TERT* promoter were amplified from HEK293T cells and cloned upstream of the *firefly luciferase* reporter gene. (**C**). **Schematic representation of the antisense TERTp-luciferase reporter plasmids.** The same PCR-amplified regions of the *TERT* promoter from HEK293T cells were cloned upstream of the *firefly luciferase* reporter gene in antisense orientation. (**D**,**E**)**. Luciferase expression in HEK293T** (**D**) **and HeLa** (**E**) **cells transfected with each of the *TERT* promoter constructs.** HEK293T (1.2 × 10^5^ cells/well) or HeLa (5 × 10^4^ cells/well) were seeded in a 48-well plate and transfected with 200 ng of each of the TERTp-luciferase reporters + HSV-TK-Renilla-luciferase for normalization (10 ng in HEK293T and 20 ng in HeLa). The promoterless *luciferase* vector was used to assess background. Cells were lysed 48 h post transfection and Firefly and Renilla Luciferase activities were measured. Firefly Luciferase relative light units (RLU) was normalized to Renilla Luciferase RLU. Results are the mean of at least 3 independent experiments carried out in duplicate or triplicate. (**F**,**G**)**. Luciferase expression in HEK293T** (**F**) **and HeLa** (**G**) **cells transfected with each of the antisense TERTp-AS-luciferase constructs.** HEK293T or HeLa cells were transfected with 200 ng of each of the Antisense TERTp-luciferase reporters + HSV-TK-Renilla-luciferase for normalization (10 ng in HEK293T and 20 ng in HeLa) in the same conditions as for panels D and E. In each experiment with TERTp-AS-luciferase vectors, the sense TERTp_−1822 to +359_-luciferase was included for comparison. Results are the mean of 3 independent experiments carried out in duplicate or in triplicate. Differences were compared using a one-way ANOVA. * indicates *p* < 0.05, ** indicates *p* < 0.01, *** indicates *p* < 0.001.

A panel of reporter vectors expressing different portions of the wild type (wt) or mutated *TERT* promoter upstream of the *firefly luciferase* gene were constructed. To that aim, a DNA fragment spanning the *TERT* promoter from position −1822 to +359 relative to the TSS was amplified from HEK293T genomic DNA (gDNA) using the following forward primer: TERTpF-1822: 5′-GAAGCTCACCCCACTCAAGTGTT-3′ and reverse primer: TERTpR+359: 5′-CACTCGGGCCACCAGCTCCTT-3′ and TA-cloned in pCR2.1 (Invitrogen, Merelbeke, Belgium). The −146C>T and −124C>T mutations were introduced in the *TERT* promoter construct individually or in combination by overlapping PCR using the same external primers and internal primers: TERT-Fmut−146: 5′-CGACCCCTT**C**CGGGTCCC-3′ and TERT-Rmut-146C>T: 5′-GGGACCCGG**A**AGGGGTCG-3′ for −146C>T and TERT- Fmut-124: 5′-CCCAGCCCC**T**TCCGGGCC -3′ and TERT-Rmut-124: 5′-GGCCCGGA**A**GGGGCTGGG-3′ for −124C>T. The mutated base is highlighted in bold. Smaller PCR fragments spanning different portions of the *TERT* promoter were amplified using different combinations of forward and reverse primers. The complete list of primers used for cloning and mutagenesis is reported in Supplementary Table S1. The following *TERT* promoter regions were amplified: *TERT* −1822 to +359 encompassing the full promoter; *TERT* −1822 to −23 spanning the core promoter and the distal and proximal upstream regulatory regions, *TERT* −499 to −23 spanning the core promoter and part of the proximal upstream promoter, *TERT* −297 to −23 spanning the core promoter, *TERT* −297 to +81 spanning the core promoter and exon 1, *TERT* −297 to +359 spanning the core promoter and the downstream regulatory region (exon 1 + intron 1 + 38 nucleotides of exon 2), *TERT* −499 to +81 spanning the proximal upstream promoter down to exon 1 and *TERT* −499 to +359 spanning the proximal upstream promoter and the downstream regulatory region. The PCR products were TA-sub-cloned into pCR2.1 and sequenced. Finally, the fragments were then cloned into pGL4.10 Luc2 (Promega, Leiden, The Netherlands) upstream of the *firefly luciferase* reporter gene in sense and antisense (AS) orientation, using Kpn I and EcoR V or Xho I and Hind III, depending on the orientation of the promoter in pCR2.1. All *TERT* promoter-*luciferase* constructs are summarized in (Figure 1A–C). The *luciferase* TSS was kept in order to assess the contribution of mutations to antisense transcription.

### 2.3. Transfection of Cells

HEK293T cells (1.2 × 10^5^/well in a 48-well plate) or HeLa cells (5 × 10^4^ cells/well in a 48-well plate) were transfected with 200 ng of each of the wt or mutated pGL4.F10Luc-*TERT* promoter-*luciferase* constructs using JetPRIME (Polyplus, via Westburg, Leusden, The Netherlands) following the manufacturer’s recommendations. To normalize for transfection efficiency, a plasmid expressing HSV-TK-Renilla-luciferase (Promega pGL4.74 hRLuc) (10 ng in HEK293T and 20 ng in HeLa cells respectively) was included in all experiments. Using 10 ng of pGL4.74 hRLuc gave high signals in HEK293T but too low signals in HeLa and thus had to be increased. For some experiments, T98G (8 × 10^4^ cells/well in a 48-well plate) and U87 (5 × 10^4^ cells/well in a 48-well plate) were used. They were transfected with 400 ng of each of the wt plasmids and 100 ng of HSV-TK-Renilla-luciferase for normalization. Lower amounts of DNA did not give exploitable Fluc or RLuc signals in these cell lines. Cells were lysed after 48 h and *firefly luciferase* induction was normalized to the corresponding Renilla Luciferase signal measured using the Dual Glo Luciferase Assay (Promega, Leiden, The Netherlands) using a Mithras LB950 luminometer (Berthold Technologies, Thoiry, France). For mutant clones, the fold-change relative to the corresponding wt construct was calculated. All transfections were performed in duplicate wells and repeated in at least 3 independent experiments for HEK293T and HeLa and two independent experiments for T98G and U87. Error bars represent standard error.

### 2.4. RNA Extraction and RT-qPCR

Total RNA was extracted from HEK293T (2 × 10^5^ cells/well), HeLa (2 × 10^5^ cells/well), T98G (2 × 10^5^ cells/well) and U87 (2 × 10^5^ cells/well) cells cultured in 24-well plates for 48 h using Trizol. One µg of total RNA was reverse transcribed with the QuantiTect reverse transcription kit (Qiagen, Venlo, The Netherlands). qPCR for lncRNA *TAPAS* and *GAPDH* was performed in duplicate using Takyon Rox dTT Blue 2X Master Mix (Eurogentec, Seraing, Belgium), and primers described in [20] or the Applied Biosystems kit for *GAPDH*. lncRNA *TAPAS* was normalized to *GAPDH* according to the 2^−^^ΔΔCt^ method [85] and relative expression levels were calculated by comparison with HEK293T cells. 

### 2.5. Statistical Analyses

Statistical analyses were performed using GraphPad Prism v5. Differences in TERT promoter-luciferase expression were compared using a one-way ANOVA followed by Newman–Keuls multiple comparison post-hoc test. Equal variance between groups was verified through Bartletts’ test. *p*-values < 0.05 were considered significant.

## 3. Results

### 3.1. Regulation of Sense and Antisense Transcription from the TERT Promoter

#### 3.1.1. Regulation of Sense Expression from the *TERT* Promoter

Most studies to date addressing the impact of mutations −146C>T and −124C>T on *TERT* reactivation have been performed using reporter vectors restrained to the core promoter [25,32,41,68,70]. Furthermore, no study has investigated why the mutations are mutually exclusive. To investigate whether transcriptional constraints underlie these observations, we engineered a panel of *firefly luciferase* reporter vectors controlled by different regions of the *TERT* promoter: the core promoter only (*TERT_core_* spanning regions −295 to −23 relative to the TSS), the core promoter with upstream regulatory regions (intermediate upstream promoter (*TERT*_−499 to −23_) or distal upstream promoter (*TERT*_−1822 to −23_), the core promoter with downstream regulatory regions (*TERT*_−295 to +81_ spanning exon 1 down to position +81 or *TERT*_−295 to +359_ spanning exon 1 + intron 1 + 38 nucleotides of exon 2, down to nucleotide +359 from the TSS), or the core promoter with both upstream and downstream regions (*TERT*_−1822 to +359_). The regulatory regions of the *TERT* promoter are shown in Figure 1A and the TERT promoter-luciferase (TERTp-luciferase) constructs are shown in Figure 1B. 

Transient transfection of the TERTp-luciferase constructs in HEK293T and HeLa cells showed high expression levels of all constructs containing the *TERT_core_* promoter alone or the core promoter and its upstream regulatory regions as long as they lacked downstream regions (Figure 1D,E). This held true for the distal upstream promoter (*TERT*_−1822 to −23_) and for the proximal upstream promoter (*TERT*_−499 to −23_). In contrast, all TERTp-luciferase constructs containing regions downstream from the core promoter, including the full *TERT* promoter (*TERT*_−1822 to +359_), led to very low or undetectable transcription levels (*p* < 0.001 in both cell lines) (Figure 1D,E). In agreement with previous reports [15,16,86], the region spanning exon 1 + intron 1 (i.e., constructs including regions down to +81 or to +359 from the TSS) nearly abrogated *TERT* expression compared with the core promoter alone (TERTp_core__-_luciferase) (*p* < 0.001 for HEK293T and HeLa cells) (Figure 1D,E) and compared with the proximal upstream (*TERT*_−499 to −295_) and distal upstream (*TERT*_−1822 to −295_) regions in HEK293T cells (*p* < 0.001). Therefore, the regions encompassing nucleotides down to position −23 relative to the TSS contain the positive regulatory elements enabling *TERT* expression, while the region spanning exon 1 + intron 1 (i.e., constructs including regions down to +81 or to +359 from the TSS) acts to negatively regulate *TERT* expression. Notably, the distal upstream 5′ region (*TERT*_−1822 to −295_) moderately decreased expression compared with the *TERT* core promoter in HeLa cells only (*p* < 0.05) (Figure 1E) to levels comparable with those reported previously [86]. Transfection of the same TERTp-luciferase constructs in two GBM cell lines carrying *TERT* promoter mutations (T98G and U87), displayed similar profiles (Supplementary Figure S1), in agreement with a previous study showing that the downstream exonic region of the *TERT* promoter inhibits *TERT* expression in a cell-independent manner [16]. Slight modulations in *TERT* expression levels between cell lines have been documented previously [16,86] and likely reflect differential expression of transcription factors between cell lines.

#### 3.1.2. Regulation of Antisense Expression from the *TERT* Promoter

The *TERT* promoter was reported to be bidirectional [15], generating a lncRNA *TAPAS* in antisense direction, which negatively regulates *TERT* expression [20]. We therefore also cloned each of the *TERT* promoter regions described above in antisense orientation upstream of the *firefly luciferase* reporter (Figure 1C) generating a mirror series of TERTp-AS-luciferase constructs. As shown in Figure 1F,G, antisense transcription was recorded for the TERTp_core_-AS-luciferase construct, in agreement with previous reports [15,20], as well as for TERTp-AS-luciferase constructs which contained downstream regions spanning exon 1, intron 1 and the first 38 nucleotides of exon 2. Of note, the TERTp_core_-AS-luciferase construct harbors the lncRNA *TAPAS* transcription initiation site, located at position −167 from the ATG, as well as the ATG from the *luciferase* gene, both of which could be used to initiate transcription. In contrast, when upstream regulatory regions were present, antisense transcription was fully repressed in both cell lines (see transcription levels of the full promoter TERTp_−1822 to +359_-AS-luciferase, TERTp_−1822 to −23_-AS-luciferase, TERTp_−499 to −23_-AS-luciferase, TERTp_−499 to +81_-AS-luciferase and TERTp_-499 to +359_-AS-luciferase) (Figure 1F,G), indicating that the region spanning the proximal upstream promoter between nucleotides −499 and −295 is responsible for inhibiting antisense transcription.

Taken together, these results confirm that the *TERT* promoter is bidirectional, that the region spanning exon 1 + intron 1 + the first 38 nucleotides of exon 2 leashes *TERT* transcription while the proximal upstream promoter (−499 to −295 from the TSS) represses antisense transcription from the *TERT* promoter.

### 3.2. TERT Promoter Mutations −146C>T and −124C>T Increase TERT Transcription in an Additive Fashion

**Figure 2:**
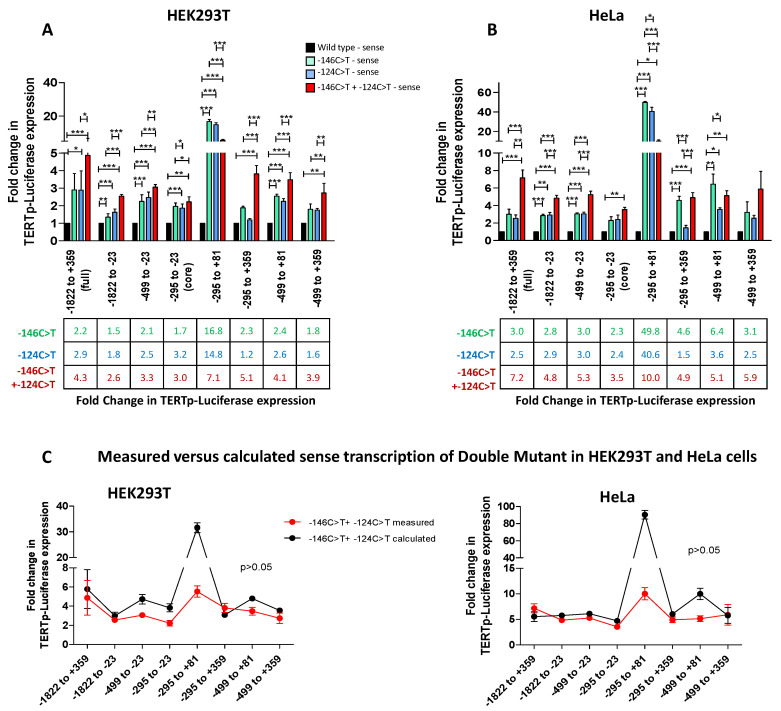
**Impact of *TERT* promoter mutations −146C>T and −124C>T on *TERT* transcription.** (**A**,**B**). Fold-change in *TERT* expression in HEK293T (**A**) and HeLa (**B**) cells transfected with the wt or mutant TERTp-luciferase reporter constructs. HEK293T (1.2 × 10^5^ cells/well) or HeLa (5 × 10^4^ cells/well) were seeded in a 48-well plate and transfected with 200 ng of each of the wt (black) or mutated (green: −146C>T; blue: −124C>T; red: −146C>T + −124C>T) TERTp-luciferase reporters + HSV-TK-Renilla-luciferase for normalization (10 ng in HEK293T and 20 ng in HeLa) for 48 h. Firefly Luciferase RLU was normalized to Renilla Luciferase RLU and transcription levels were further normalized to the wt for each *TERT* promoter construct. Results are the mean of at least 3 independent experiments carried out in duplicate. Error bars represent standard error. Differences were compared using a one-way ANOVA followed by a Newman–Keuls post-hoc test for pairwise comparisons. * indicates *p* < 0.05, ** indicates *p* < 0.01, *** indicates *p* < 0.001. (**C**). Comparison of the measured and estimated fold increase in *TERT* expression for the double mutant. The fold increase in TERTp-luciferase expression measured for the double mutant (red) was compared with the calculated sum of the fold increase due to the −146C>T mutation + −124C>T mutation (black).

To assess the impact of the two main *TERT* promoter mutations −146C>T + −124C>T on *TERT* transcription, we introduced the mutations alone and in combination in each of the sense TERTp-luciferase reporter vectors, generating 4 constructs for each *TERT* promoter region: wt, −146C>T mutant, −124C>T mutant and −146C>T + −124C>T (double mutant). As shown in Figure 2A, the −146C>T mutation increased *TERT* promoter-driven expression by 1.5 to 2.4-fold and the −124C>T mutation by 1.6 to 3.2-fold in HEK293T cells (Figure 2A). This increase reached statistical significance in most cases. Similarly in HeLa cells, the −146C>T increased transcription by at least 3-fold and the −124C>T mutation by >3-fold with statistical support (Figure 2B). Despite variations between cell types, the increase in *TERT* promoter-driven luciferase was recorded consistently for most constructs. Furthermore, there was no statistical difference in the increase mediated by −146C>T and −124C>T. Of note, when expressed in the TERTp_−295 to +81_-luciferase construct, the single mutations boosted expression by over 15-fold in HEK293T cells and by over 40-fold in HeLa cells (*p* < 0.001 in both cases) (Figure 1A,B). Since the first exon covered by this promoter fragment was described to be epigenetically regulated and to contain a binding site for CTCF [16], this result suggests that the presence of either of the two mutations might affect the binding of transcriptional repressors. Yet, although TERTp-luciferase was increased by each of the mutations, this trend did not reach statistical significance when the region encompassing exon 1 + intron 1 + 38 bp of exon 2 was present, in both HEK293T cells and in HeLa cells, illustrating the strong transcriptional repression exerted by intron 1 + exon 1 + 38 bp of exon 2.

The double mutant enabled significantly higher transcription levels compared with the wt *TERT* promoter (*p* < 0.01 or *p* < 0.001) and compared to each of the single mutants for all constructs in both cell lines (Figure 2A,B). Generally, the two mutations together showed an additive increase in TERT expression levels (Figure 2C). This finding held true for all constructs which included upstream distal or proximal regulatory regions and for constructs containing the downstream regulatory region spanning exon 1 + intron 1 + the first 38 nucleotides of exon 2. For the TERTp_core_-luciferase construct, a similar trend was recorded, although statistical support was reached only in HEK293T cells for the −146C>T mutant compared to the double mutant (Figure 2A,B), suggesting that the transcriptional advantage linked to the two mutations involves elements contained in the upstream regulatory regions. Intriguingly, the two constructs which included the first exon (TERTp_−295 to +81_-luciferase and TERTp_−499 to +81_-luciferase), had similar or lower transcription when the two mutations were present compared with the single mutants (Figure 2A–C). While the single mutations individually relieve the binding of inhibitory factors to exon 1, the two mutations concomitantly may lead to unfavorable chromatin looping or structures that ultimately decrease transcription over the single mutations. Overall, our results indicate that the double mutant confers a transcriptional advantage over the single mutants and argue against a transcriptional constraint being the main reason for the mutual exclusion of *TERT* promoter mutations in vivo.

### 3.3. TERT Promoter Mutations Increase Antisense Transcription from the TERT Promoter

**Figure 3:**
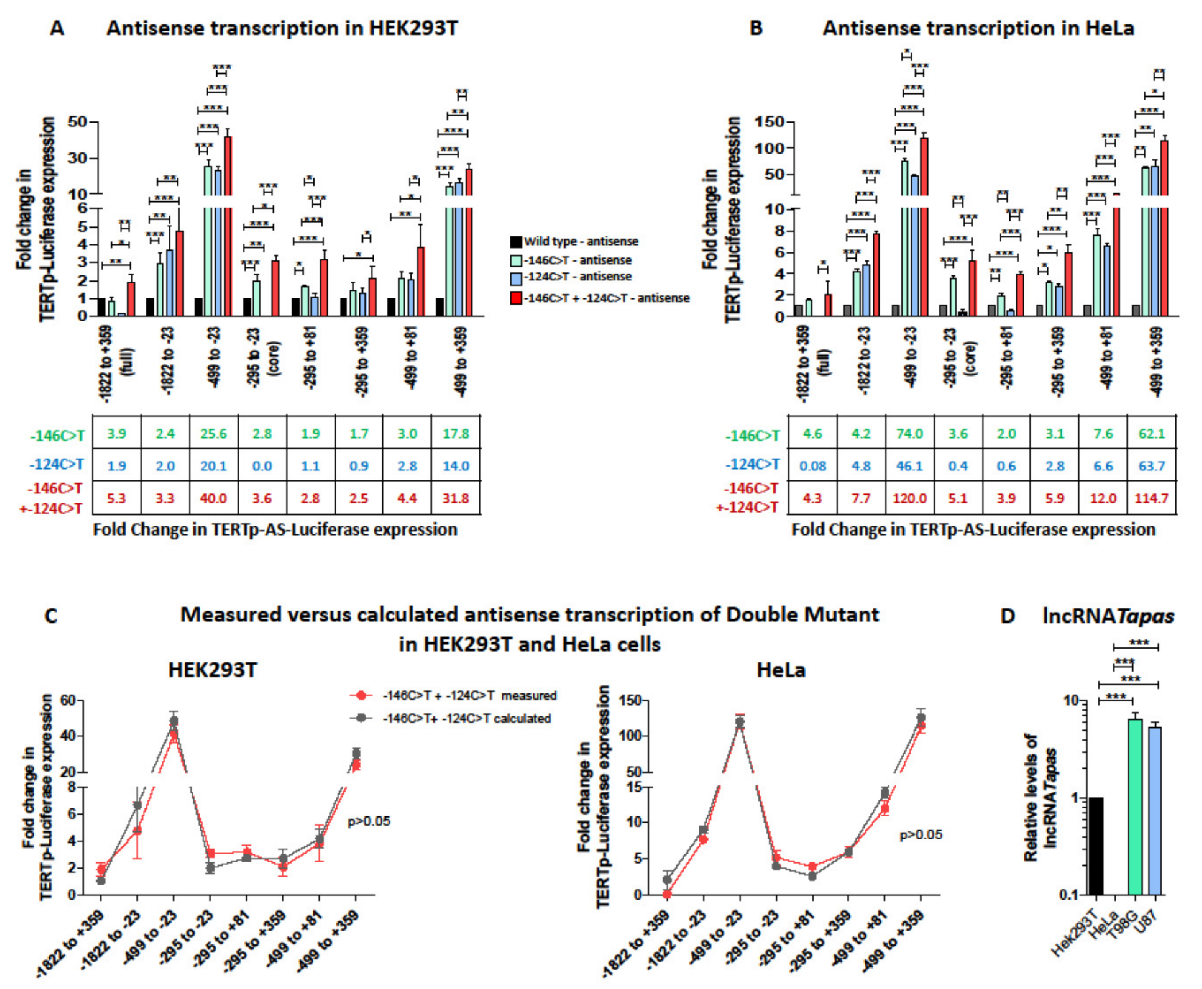
**Impact of *TERT* promoter mutations** −**146C>T and** −**124C>T on antisense expression from the *TERT* promoter.** (**A**,**B**). Fold-change in *TERT* expression in HEK293T (**A**) and HeLa (**B**) cells transfected with the wt or mutant TERTp-luciferase reporter constructs. HEK293T (1.2 × 10^5^ cells/well) or HeLa (5 × 10^4^ cells/well) were seeded in a 48-well plate and transfected with 200 ng of each of the wt (black) or mutated (green: −146C>T; blue: −124C>T; red: −146C>T + −124C>T) TERTp-AS-luciferase vectors + HSV-TK-Renilla-luciferase for normalization (10 ng in HEK293T and 20 ng in HeLa) for 48 h. Firefly Luciferase RLU was normalized to Renilla Luciferase RLU and transcription levels were further normalized to the wt for each *TERT* promoter construct. Results are the mean of at least 3 independent experiments carried out in duplicate. Error bars represent standard error. (**C**). Comparison of the measured and estimated fold increase in *TERT* antisense expression for the double mutant. The fold increase in TERTp-AS-luciferase expression measured for the double mutant (pink) was compared with the calculated sum of the fold increase due to the −146C>T mutation + −124C>T mutation (grey). (**D**). Relative expression of lncRNA *TAPAS* in different cell lines. Total RNA was extracted from cells (2 × 10^5^ cells) cultured for 48 h and 1 μg RNA was reverse transcribed. Relative levels of lncRNA *TAPAS* were compared in HEK293T, HeLa, T98G (−146C>T) and U87 (−124C>T) cells using the 2^−^^ΔΔCt^ method and relative expression levels were calculated by comparison with HEK293T cells. Experiments were performed in duplicate wells and the mean +/− standard error of two independent experiments are represented. Differences were compared using a one-way ANOVA followed by a Newman–Keuls post-hoc test for pairwise comparison. * indicates *p* < 0.05, ** indicates *p* < 0.01, *** indicates *p* < 0.001.

Next, we investigated whether *TERT* promoter mutations impacted antisense transcription from the *TERT* promoter, introducing the −146C>T and −124C>T mutations alone or together in each of the TERTp-AS-luciferase constructs. As shown in Figure 3A,B, antisense transcription from the full *TERT* promoter (*TERT*_−1822 to +359_) and from the *TERT_core_* promoter was upregulated by the −146C>T mutation, but not by the −124C>T mutation in both cell lines, suggesting that the latter mutation only impacts sense transcription. In the presence of downstream regulatory elements (exon 1 only or exon 1 + intron 1 + 38 bp of exon 2), the −124C>T slightly increased antisense transcription compared with the wt promoter, but less than the −146C>T. However, when the upstream regulatory elements were present, antisense transcription was increased comparably by both mutations, to levels ranging from 2-fold to 63-fold (Figure 3A,B). Remarkably, the transcriptional increase was particularly striking in constructs including only the proximal upstream regulatory sequences and lacking the distal upstream regulatory sequences (i.e., downstream from position −499 from the TSS), while it ranged from 2- to 4.6-fold only when the distal upstream regulatory sequences were present. This observation suggests that repressor elements which are not affected by *TERT* promoter mutations bind in the distal upstream promoter region. The presence of the first exon also mitigated the impact of *TERT* promoter mutations on antisense transcription to an intermediate level, perhaps due to occupational interference. Overall, since the full *TERT* promoter (TERTp_−1822 to +359_-AS-luciferase), which harbors both upstream and downstream regulatory elements, and the *TERT_core_* promoter consistently show upregulation of antisense transcription by the −146C>T mutation only, it is likely that in vivo the −146C>T mutation more efficiently allows an overcoming of the repression of antisense transcription.

As for sense transcription, the double mutant led to an additive transcriptional increase in both cell lines (Figure 3C). In line with this finding, in constructs where only −146C>T had an impact, the double mutant had a similar impact to the −146C>T single mutant.

Next, we assessed whether the increase in antisense transcription from the *TERT* promoter by *TERT* promoter mutations translates into increased lncRNA *TAPAS* levels. To that aim, we measured lncRNA *TAPAS* levels in two GBM cell lines harboring the monoallelic −146C>T (T98G) and −124C>T (U87) *TERT* promoter mutations. We detected much lower levels of lncRNA *TAPAS* in HeLa than in HEK293T cells, again highlighting cell-type specificities, and significantly higher levels of lncRNA *TAPAS* in cells with *TERT* promoter mutations compared with HEK293T and HeLa (Figure 3D). Because there are no cell lines harboring the two mutations, we could not measure lncRNA *TAPAS* levels expressed by a promoter harboring the two mutations.

Together, these results show that sense and antisense transcription from the *TERT* promoter are linked, and that the −146C>T mutation upregulates both *TERT* expression and antisense transcription, generating lncRNA *TAPAS*. Therefore, while *TERT* transcription is increased by C-146C>T, the concomitant increase of lncRNA *TAPAS* may tame TERT levels in a negative feedback loop. As lncRNA *TAPAS* levels are inversely correlated with TERT levels, it is tempting to hypothesize that higher lncRNA *TAPAS* associated with the −146C>T mutation may explain the higher prevalence of −124C>T in vivo. In this scenario, the two mutations concomitantly would increase lncRNA *TAPAS* levels similarly or even further, ultimately mitigating the advantage of a single *TERT* promoter mutation. Physiologically, this dual regulation may help keep TERT levels below a given threshold.

## 4. Discussion

In this study, we investigated the impact of the two most common *TERT* promoter mutations, alone or in combination, on sense and antisense transcription from the *TERT* promoter. Our aim was to assess whether a transcriptional constraint could explain why *TERT* promoter mutations are mutually exclusive. In line with previous reports based on the *TERT* core promoter [23,25,26,27,28,29,31,41,67,68,69], we confirm that the mutations increase *TERT* transcription levels from the core promoter by 2–6-fold and that both mutations increase transcription similarly. We extend these findings by showing that overall the transcriptional increase was comparable across most constructs, regardless of whether they include upstream or downstream regulatory elements (Figure 2). This observation suggests that the presence of *TERT* promoter mutations is sufficient to overcome the transcriptional silencing of *TERT* in normal cells. The fact that the two mutations led to a comparable increase in sense transcription argues against the hypothesis that a transcriptional advantage selects for the overwhelming predominance of the −124C>T mutation in vivo. One possible explanation could be that this mutational bias reflects different aetiologies. The cytosine located −124 bp upstream from the TSS could be more accessible to enzymes, including APOBEC3 enzymes, and may therefore be more easily deaminated. In specific malignancies such as skin cancers, in contrast, the two mutations are equally frequent [42,46,47] and may be caused by UV radiation and C>T mutations at CC dinucleotides. Therefore, the differential distribution of *TERT* promoter mutations across cancer types may reflect different aetiologies, APOBEC3- and aging-induced deamination on one hand, versus UV-induced mutations on the other [21].

Our findings further reveal that the −146C>T mutation also unleashes antisense transcription from the *TERT* promoter (Figure 3A,B) and that lncRNA *TAPAS* is higher in prototypical cell lines harboring monoallelic TERT promoter mutations (Figure 3D). In a previous study, Malhotra et al. had recorded no impact of the −124C>T mutation on antisense transcription driven by the *TERT* core promoter [20]. Our results confirm that −124C>T does not affect antisense transcription from the *TERT* promoter but importantly, they reveal that the −146C>T mutation consistently reactivates antisense transcription from the *TERT* promoter, be it restricted to the *TERT* core promoter or inclusive of upstream and downstream regulatory elements (Figure 3). Moreover, we show that both mutations upregulate antisense transcription to equivalent levels when upstream regulatory elements only are present. The fact that the −124C>T mutation decreases antisense transcription from the *TERT_core_* promoter compared with the wt and to all other constructs suggests that the transcription factor that binds the novel GGAA site at position −124 needs to dimerize and thus concomitantly binds to another GGAA site beyond the boundaries of the core promoter. One candidate could be GABPA [70]. NF-κB is a less likely candidate as it preferentially binds the −146C>T [69]. In this case, binding to −124C>T would be possible when downstream or proximal upstream regulatory sequences are present. However, when all regulatory elements are present (*TERT*_−1822 to +359_), this binding site seems to no longer be exploited or accessible. One possible reason could be DNA looping resulting in ternary DNA structures that do not allow the binding of transcription factors to GGAA at position −124 and even at neighboring GGAA sites. In this scenario, the –146 position would, *a contrario*, still remain accessible, and thus not be impacted by the DNA looping. Alternatively, access to RNA Pol II to the antisense transcriptional initiation site may be affected by the ternary structure adopted by DNA in the presence of the −124C>T mutation. Answers to these questions warrant further investigation. These observations suggest that the −146C>T mutation upregulates antisense transcription and thereby lncRNA *TAPAS* concomitantly to *TERT*, and might thus negatively affect TERT levels. How lncRNA *TAPAS* impacts TERT levels remains elusive, as only an inverse correlation has been described to date [20]. Yet, it is tempting to hypothesize that higher lncRNA *TAPAS* decrease TERT and ultimately contribute to the selective advantage of −124C>T and its strong overrepresentation across cancers. In this study, we only investigated the effect of the two most frequent *TERT* promoter mutations, −146C>T and −124C>T. Other mutations which create novel Ets/TCF binding sites pepper the *TERT* promoter [44,49]. They all have similar effects on *TERT* transcription [23,25,26,27,28,29,31,32,41,42,53,67,68,69,87,88]. Since they are much less prevalent than the −124C>T and −146C>T mutation, it is possible that they may also increase antisense transcription. Further studies would be needed to verify the impact of these mutations on antisense transcription and on lncRNA *TAPAS* levels.

The strongest impact of both *TERT* promoter mutations, alone or in combination, on antisense transcription was recorded for constructs containing the proximal upstream promoter region appended to the *TERT* core promoter in both cell lines (Figure 3A,B, compare TERTp_core_-AS-luciferase to TERTp_−499 to −23_-AS-luciferase or TERTp_−295 to +359_-AS-luciferase to TERTp_−499 to +359_-AS-luciferase). This region hosts binding sites for strong repressors such as CTCF [15,16], and is known to negatively regulate antisense transcription from the *TERT* promoter ([15,17] and Figure 1F,G). It also overlaps the THOR sequence, which contains 52 CpG islands and is subject to epigenetic modifications which affect *TERT* sense expression [15,17,18,19]. Our results pinpoint this region spanning nucleotides −499 and −295 from the TSS as the region most responsive to derepression by *TERT* promoter mutations and suggest that the −146C>T mutation may relieve the binding of these repressors. The distal upstream *TERT* promoter region tames this upregulation and thus further contributes to negatively regulate antisense transcription from the *TERT* promoter (Figure 3A,B, compare TERTp_−499 to −23_-AS-luciferase to TERTp_−1822 to −23_-AS-luciferase or TERTp_−499 to +359_-AS-luciferase to TERTp_−1822 to +359_-AS-luciferase), but its impact is much less directly affected by the presence of *TERT* promoter mutations. Together, these results suggest that repressors binding to the region encompassing the proximal upstream regulatory region (−499 to −295 from the TSS) can be displaced by transcription factors such as GABPA or Ets/TCF binding to mutations in the *TERT* promoter, while the distal upstream regulatory region is less affected by such a mechanism. It is noteworthy that epigenetic tuning of transcription cannot be investigated using TERTp-luciferase reporters, and only the balance between activator and repressor transcription factors is measured using this system. Further studies would be needed to ascertain whether methylation patterns and lncRNA *TAPAS* expression are affected by *TERT* promoter mutations.

Most interestingly, when the two mutations were expressed together, the increase in *TERT* sense and antisense transcription was significantly higher than the increase enabled to the single mutations. Furthermore, it corresponded to the additive effect of each of the single mutations alone (Figure 2C and Figure 3C). Therefore, the double mutant would *a priori* provide a direct transcriptional advantage over the single mutants. Yet, the higher transcription from the double mutant *TERT* promoter may also increase antisense transcription driven by the −146C>T mutation and thereby increase levels of lncRNA *TAPAS*. As lncRNA *TAPAS* and *TERT* expression are negatively correlated, lncRNA *TAPAS* has been proposed to act as a negative regulator of *TERT* expression in a negative feedback loop [20]. High antisense transcription mediated by −146C>T mutation could thus increase lncRNA *TAPAS* levels and thereby lead to an overall decrease in *TERT* expression in a negative feedback loop. Such a self-regulatory mechanism could explain the mutual exclusion of *TERT* promoter mutations in vivo. We could not measure the impact of both mutations on lncRNA *TAPAS* levels because there are no cell lines harboring both mutations concomitantly.

*TERT* promoter mutations are generally mutually exclusive with each other [41] as well as with other telomere maintenance mechanisms such as copy number duplications [79,80], viral oncogenes [21,53,54,55,66,67,79,81,82,83,84] or alternative lengthening of telomeres (ALT) [22,33,34,57]. The reasons for this strict exclusion remain speculative to date but it is thought that it reflects a narrow window in terms of TERT homeostasis. Indeed, TERT levels are exquisitely balanced [21]. *TERT* promoter mutations generally occur late in tumor genesis, after the appearance of driver mutations in other oncogenes such as RAS/BRAF, EGFR, which lead to constitutive cell division and thus accelerate telomere erosion. *TERT* reactivation regenerates telomeres in cells with critically short telomeres sufficiently to maintain them above the critical threshold and to stabilize the tumor genome, thereby maintaining self-renewal potential [3,89,90,91,92]. Moreover, TERT also exerts other functions which are unrelated to telomere elongation but alter cell turnover. It contributes to cell survival and proliferation by facilitating Wnt/β-catenin-dependent [93,94], c-Myc-dependent [95,96], and NF-κB-dependent gene transcription [97,98], thereby sustaining both oncogenic signaling pathways and its own transcription in a feedforward loop [99,100]. The proliferative advantage conferred by *TERT* reactivation however also has a cost in terms of metabolic alterations, oxygen and nutrient supplies and the tumor microenvironment. A modest increase in *TERT* expression (2- to 4-fold) and a single genetic mechanism of telomere elongation enable sufficient telomere healing to avoid telomere crisis and cell death [11,22,32,39,57,60,100,101], yet remain sustainable in terms of oxygen and nutrient supplies. TERT homeostasis is thus likely an exquisite balance between escape from apoptosis resulting from telomere attrition and genomic instability on one hand, and cell sustainability in terms of oxygen and nutrient supplies on the other. In line with this view, it is remarkable that *TERT* promoter mutations are generally found in solid cancers with relatively low self-renewal rate [22], while they are rare (<15%) in cancers with high turnover and intrinsic *TERT* expression, such as hematopoietic, lymphoid, or gastrointestinal malignancies [3,22,32,80,102]. We suggest a model whereby *TERT* promoter mutations increase both sense (*TERT*) and antisense (lncRNA *TAPAS*) transcription to maintain TERT protein levels within a sustainable threshold. Disproportionate levels of one transcription product would shift the balance towards insufficient or excessive TERT, eventually leading to cell death by apoptosis or starvation. Such a self-regulatory mechanism where *TERT* promoter reactivation promotes *TERT* expression together with a *TERT* auto-regulatory mechanism is an extremely elegant means to ensure this equilibrium is maintained. Further studies will be needed to assess whether lncRNA *TAPAS* is the only mechanism ensuring *TERT* transcriptional homeostasis.

## Supplementary Materials

The following are available online at https://www.mdpi.com/article/10.3390/biomedicines9121773/s1, Table S1: List of primers used for *TERT* promoter cloning and mutagenesis. Table S2: List of primers used for qRT-PCR. Supplementary Figure S1: *TERT* promoter-driven transcription in T98G and U87 GBM cell lines.
