# Supplementary material for: TERT Promoter Mutations Increase Sense and Antisense Transcription from the TERT Promoter
Source: Biomedicines. 2021 Nov 26;9(12):1773. doi: 10.3390/biomedicines9121773 (PMC8698883; doi:10.3390/biomedicines9121773)
Supplement: Supplementary file 1 [file biomedicines-09-01773-s001.zip › biomedicines-1433041-supplementary.pdf]

Supplementary Materials

Supplementary Table S1: List of primers used for *TERT* promoter cloning and mutagenesis

	**Forward Primer**	**Reverse Primer**	**application**	**Ref**
TERTpF -1822	5'-GAAGCTCACCCCACTCAAGTGTT-3'		cloning	
TERTpF -499	5'-CCAAAGGGTCGCCGCACGCAC-3'		cloning	[1]
TERTpF -295	5'-CAGGCCGGGCTCCCAGTGGATT-3'		cloning	
TERTpF -23		5'-GGCTTCCCACGTGCGCAGCAGGA-3'	cloning	[1]
TERTpR +81		5'-GAACGTGGCCAGCGGCAGCACCT-3'	cloning	[1]
TERTpR +359		5'-CACTCGGGCCACCAGCTCCTT-3'	cloning	
TERTpmut -146C>T	5'-CGACCCCT**T**CCGGGTCCC-3'	5'-GGGACCCGG**A**AGGGGTCG-3'	mutagenesis	
TERTpmut -124C>T	5'- CCCAGCCCC**T**TCCGGGCC -3'	5'-GGCCCGGA**A**GGGGCTGGG-3'	mutagenesis	

Supplementary table S2. List of primers used for qRT-PCR and qPCR

**lncRNA*TAPAS***	5'-TGAGCAACCACCCCAAATCT-3'	5'-TTTCCCACCCTTTCTCGACG-3'	[2]
**GAPDH**	5'-GAAGGTGAAGGTCGGAGTC-3'	5'-GAAGATGGTGATGGGATTTC-3'	

1. Renaud S, Bosman FT, Benhattar J. Implication of the exon region in the regulation of the human telomerase reverse transcriptase gene promoter. Biochem Biophys Res Commun. 2003;300(1):47-54. Epub 2002/12/14. PubMed PMID: 12480519.

2. Malhotra S, Freeberg MA, Winans SJ, Taylor J, Beemon KL. A Novel Long Non-Coding RNA in the hTERT Promoter Region Regulates hTERT Expression. Noncoding RNA. 2017;4(1). Epub 2018/04/17. doi: 10.3390/ncrna4010001. PubMed PMID: 29657298; PubMed Central PMCID: PMCPMC5890388.

Supplementary Figure S1. ***TERT* promoter-driven transcription in T98G and U87 GBM cell lines**

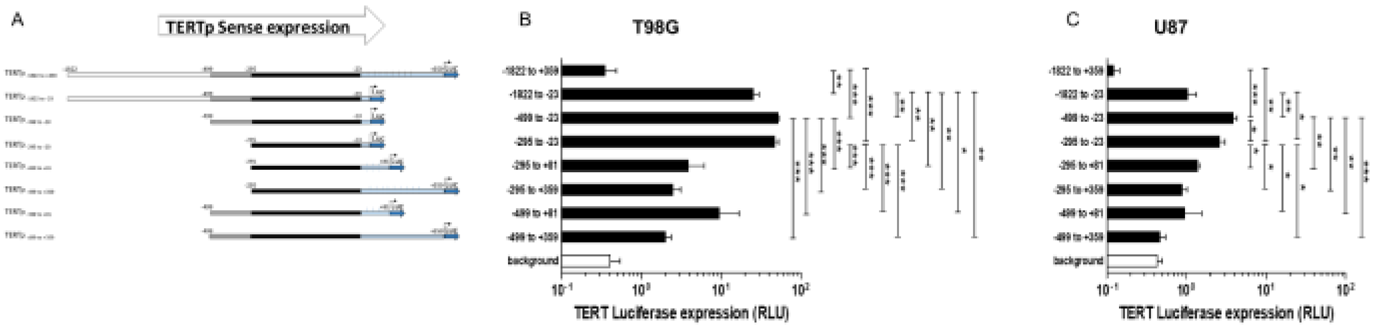

A. Schematic representation of the TERTp-luciferase reporter plasmids in sense orientation. B and C. *TERT* promoter-driven *luciferase* expression in T98G (B) and U87 (C) cells. T98G ($8x10^4$ cells/well) or U87 ($5x10^4$ cells/well) were seeded in a 48-well plate and transfected with 400 ng of each of the TERTp-luciferase vectors + 100 HSV-TK-Renilla-luciferase for normalization. Cells were lysed 48 hours post transfection and Firefly- and Renilla-Luciferase activities were measured. Firefly-Luciferase RLU was normalized to Renilla-Luciferase RLU. Results are the mean of 2 independent experiments carried out in duplicate wells. Error bars represent standard error. Differences were compared using a one-way ANOVA followed by a Newman-Keuls post-hoc test for pairwise comparisons. * indicates p<0.05, ** indicates p<0.01, *** indicates p<0.001.
